# Pituitary gland metastasis from rectal cancer: report of a case and literature review

**DOI:** 10.1186/2193-1801-2-467

**Published:** 2013-09-16

**Authors:** Margherita Ratti, Rodolfo Passalacqua, Rossana Poli, Enrico Betri, Mario Crispino, Roberto Poli, Gianluca Tomasello

**Affiliations:** Oncology Division, Azienda Istituti Ospitalieri di Cremona, Viale Concordia 1, Cremona, 26100 Italy; Pathology Division, Azienda Istituti Ospitalieri di Cremona, Cremona, Italy; Radiology Division, Azienda Istituti Ospitalieri di Cremona, Cremona, Italy; Neurosurgery Division, Azienda Istituti Ospitalieri di Cremona, Cremona, Italy

**Keywords:** Pituitary, Metastasis, Rectal cancer, Chemo-radiotherapy

## Abstract

Pituitary metastases are unusual complications of malignancies. In about only 2% of patients they origin from colorectal cancer (CRC), with breast and lung as the most common primary tumors. Nevertheless, some authors reported a recent increase of the incidence of metastases in infrequent sites, such as brain or bone, arising from gastrointestinal cancers, probably due to the expanded treatment options and the resulting improved survival. Here, we report the case of a 54-year old woman diagnosed with lung metastases from rectal cancer, who, after several cycles of radio- and chemotherapy, presented symptoms and signs of pituitary disfunction (i.e. diabetes insipidus, hypothyroidism and diplopy). The diagnosis of pituitary metastasis from rectal cancer was histologically confirmed after surgery.

## Introduction

Metastases to the pituitary gland are rare complications of advanced cancer. Data from a large number of autopsies show that pituitary metastases are present in 1–3.6% of patients with malignancies (McCormick et al. 1989). Breast cancer is the most common tumor metastasizing to the pituitary gland, followed by lung, prostate, renal cell and gastrointestinal cancers; lymphoma, leukemia, thyroid carcinoma, and plasmocytoma have also been described (Fassett and Couldwell 2004). Brain metastases from CRC are very uncommon; in a recent analysis a cumulative incidence of 1.2% was reported. In particular, CRC is a rare cause of metastases to the pituitary gland (Schouten et al. 2002). In a series of 380 cases it was the primary tumor in only 2.4% of such cases (9 patients) (Komninos et al. 2004). Before the advent of modern systemic therapies most CRC patients lived for only few months. Late onset metastases in brain or central nervous system rarely developed and patients died before such metastases could occur. Recent therapies allow longer disease-free intervals and better overall survival; this probably explains the current increase in the incidence of metastases from colorectal cancers in uncommon sites, such as brain and/or bone (Go et al. 2011). Here we report an unusual case of a young-adult woman, who developed pituitary metastases from rectal cancer 4 years after the resection of the primary tumor and following prior neo- and adjuvant treatments.

### Case description

A 54-year old caucasian woman was admitted to the radiotherapy division of our hospital in September 2007, with new diagnosis of adenocarcinoma of the rectum (clinical stage T3, N2, M1 [lung]). The patient, after neoadjuvant radio-chemotherapy (50 Gy totally delivered to the posterior pelvis from September 27th to November 16th associated with Oxaliplatin 75 mg/mq weekly + 5FU 225 mg/mq as a continuous intravenous infusion from September 27th to October 5th, early interrupted after 2 weeks for severe diarrhea and fever), underwent an abdomino-perineal resection of the rectum in January 2008, followed by adjuvant chemotherapy with FOLFOX4 scheme (12 cycles, from February 27th to September 3rd). A CT (computed tomography) scan of her chest revealed a size reduction of the two pulmonary lesions in September 2008.

Subsequent controls (TC and PET scans) showed pulmonary progression of disease and tumor markers increase (i.e. CEA), without any evidence of abdominal relapse in July 2009. The patient agreed to start a new line of chemotherapy in April 2010 only, after a new CT scan carried out in March 2010 showed further increase in size of the pulmonary lesions. Irinotecan 200 mg/m2 and Bevacizumab 7.5 mg/Kg every 3 weeks were administered from April 9th to June 7th (Irinotecan early stopped for gastrointestinal toxicity and patient’s refusal) followed by Bevacizumab alone at a dose of 5 mg/Kg bi-weekly for ten cycles from July until December, with instrumental evidence of stable disease until January 2011, when a CT of her chest and a PET scan revealed a new progression of disease in this site. For this reason, she started a new treatment with Raltitrexed 3 mg/mq q3w in January 2011, early interrupted after 1 cycle for severe bone marrow toxicity.

The patient decided to refuse any further treatment until April 2011, when she was admitted to the Oncology division of our hospital asking for second medical opinion and, after an instrumental restaging, she was enrolled in a clinical trial in May 2011. Because of her wild-type k-ras mutational status, she received Cetuximab 361 mg weekly, for 8 cycles until July 2011, when a restaging CT scan showed the appearance of a new pulmonary lesion. Therefore, patient was offered a new chemotherapy with XELOX scheme (Oxaliplatin 141 mg, day 1, Capecitabine 2500 mg day 2–15 q3w) in August 2011, suspended after the 3^rd^ cycle for moderate neurotoxicity and severe diarrhea. The patient underwent an instrumental re-evaluation with thoraco-abdominal CT scan in October 2011, which evidenced dimensional increase of pulmonary lesions.

Taking into consideration the previous chemotherapy toxicities and the patient’s reluctance to receiving new potentially toxic agents, we offered a palliative treatment with cyclophosphamide and methotrexate at low continuative doses, which patient started in November 2011. The following CT scan revealed stable disease in February 2012, but the patient decided to interrupt treatment for severe asthenia and mucositis, until April 2012, when, we decided to administer Mitomycin C at the dose of 6 mg/m2 every 4 weeks, after the evidence of further increase in size of the pulmonary lesions.

The patient, after the third cycle of therapy, complained poliuria and polidipsia, but there was no evidence of plasmatic and urinary osmolites alteration. For this reason, we planned a restaging CT, including brain imaging.

**Figure 1:**
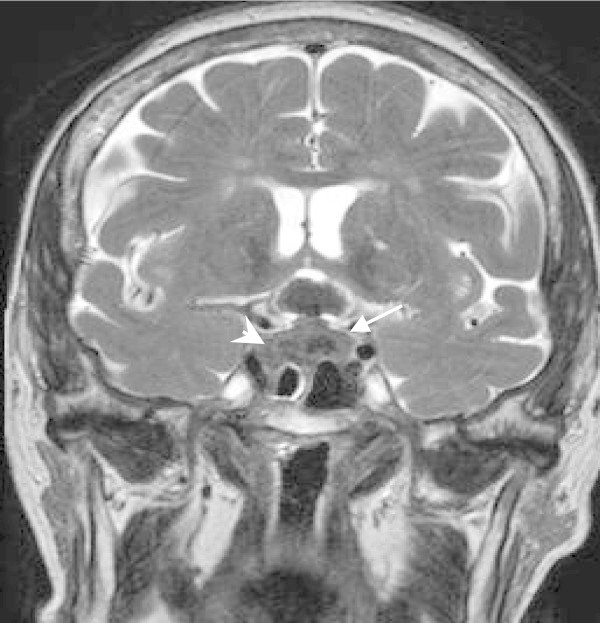
**Coronal T2WI MR.** Intra and suprasellar “snowman-shaped” mass showing mixed intensity signal with hypointense focus of hemorrhage present within the lesion (arrow). The tumor invades the cavernous sinus (arrowhead).

**Figure 2:**
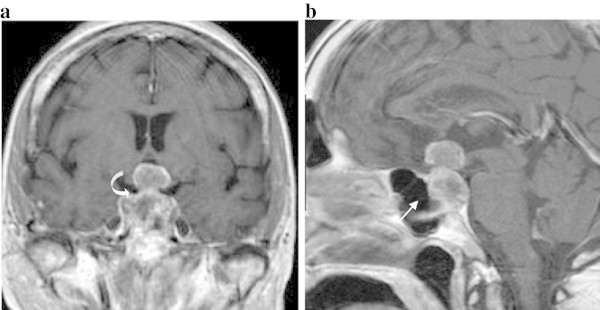
**Coronal (A) and Sagittal (B) T1WI MR after contrast medium administration.** Large pituitary mass with inhomogeneous enhancement involving the infundibular stalk (curved arrow) and extending into suprasellar cistern. The mass expands and deepens the bony sella (arrow).

**Figure 3:**
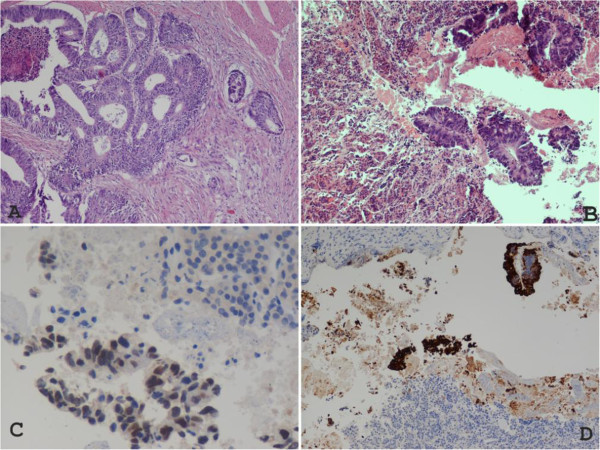
**Histological examination of the pituitary lesion surgically removed. A:** Primary site of disease (colorectal tissue; hematoxylin-eosin stain). **B:** Adenohypophyseal parenchimal tissue with evidence of metastatic cells from adenocarcinoma. On the left side: Adenohypophyseal tissue; On the right side: Clusters of neoplastic glandular cells (hematoxylin-eosin stain). **C:** Metastatic adenocarcinomatous cells invading adenohypophyseal parenchimal tissue. No evidence of immunocoloration for CDX-2 (caudal type homebox transcription factor 2) into adenohypophyseal parenchimal tissue (upper right corner of the image); positive nuclear CDX-2 immunocoloration found in clusters of metastatic adenocarcinoma cells agreeing with the rectal origin of the lesion. **D:** Metastatic adenocarcinomatous cells invading adenohypophyseal parenchimal tissue. No evidence of immunocoloration for cytokeratin 20 into adenohypophyseal tissue; positive cytokeratin 20 immunocoloration found in clusters of metastatic adenocarcinomatous cells.

Few days later, she was admitted to the Neurosurgery Unit reporting, in addition to polidipsia and poliuria, recurrent episodes of headache and vomit, associated with diplopy, decrease in visual acuity and ophtalmoplegy at the right eye. Brain CT scan and magnetic resonance imaging revealed the presence of a voluminous endo-sovrasellar lesion (cranio-caudal diameter: 25 mm; trasversal diameter: 20 mm), compressing the optic chiasm extended to the third ventricle and to both cavernous sinuses, in particular to the right one (Figures 1 and 2). Hyponatremia (126 mEq/L; normal range 135–145 mEq/L), low urine specific weight (1001; normal range 1014–1030), subclinical hypothyroidism (TSH 0.0010 μUI/ml; normal range 0.400-4.200 μUI/ml), were also found, confirming the diagnosis of pituitary failure (central diabetes insipidus and central hypothyroidism). In order to improve the patient’s clinical conditions (diabetes insipidus, in particular), therapy with desmopressin acetate, 1 puff twice a day in both nostrils was started. This therapy promptly improved the symptoms of diabetes insipidus (we observed decrease of polydipsia and polyuria with normalization of urine specific weight in 4 days) but the severe diplopia and other visual field impairments such as hemianopsia, cephalea and vomit seriously compromised patient’s quality of life. Therefore, the lesion was surgically removed and histological examination confirmed the diagnosis of adeno-carcinoma of intestinal origin (Figure 3).

Despite the improvement of neurological symptoms after surgery, the patient died of respiratory complications as a result of the lung disease involvement 8 days later, in August 2012.

## Discussion

The pituitary gland is an uncommon site for metastases, in particular from CRC. Breast and lung cancers are the most common diseases spreading to this site (Morita et al. 1998). Teears and Silverman analysed 88 patients with pituitary metastases and noticed that breast and lung were the most frequent sites of the primary tumor (40% and 33%, respectively), whereas 2% of patients only presented CRC as primary site of disease (Teears and Silverman 1975). In the absence of specific series in the literature, the incidence of rectal cancer metastasizing to the pituitary is certainly even lower.

The widest review of literature about pituitary metastases including 380 patients, mentions 9 cases only of primary localization in colorectal site (Komninos et al. 2004). These data confirm the rarity of our report.

Recent studies reveal that the majority of pituitary metastases occur in the posterior lobe. The same work by Teears and coll., reported that 57% of the lesions are localized to the posterior pituitary alone, 13% to the anterior pituitary alone, 12% to both lobes and the remaining to the capsule or stalk (Teears and Silverman 1975).

The majority of pituitary metastases are clinically silent. When the patient is symptomatic, the most common clinical presentation seems to be diabetes insipidus (DI) (45.2%), reflecting a predominance of metastases to the posterior lobe (Sioutos et al. 1996). Other reported symptoms are ophtalmoplegia, headache, visual field defects and anterior pituitary disfunction (Nelson et al. 1987). Additionally, DI is more common in patients with pituitary metastases than in those with adenomas (Morita et al. 1998). In a series of 190 cases of symptomatic pituitary metastasis, DI was reported in 45.2% of patients, optic nerve impairment in 27.9% and anterior pituitary insufficiency in 23.6% (Komninos et al. 2004). Twenty-one percent of patients developed other cranial nerves defects such as 3^rd^, 4^th^, and 6^th^ palsy.

In about 25% of the cases the diagnosis is made after laboratory evidence of anterior pituitary failure (Freda and Post 1999).

Radiological evaluation doesn’t really help in distinguishing adenoma from metatasis. High resolution CT and MRI are the most sensitive exams. CT usually shows a hyperdense or isodense mass, enhancing homogeneously or non-homogeneously (if cystic degeneration, hemorrhage, or necrosis co-exist) in contrast images. MRI may demonstrate an isointense or hypointense mass on T1 weighted images, with a usually high-intensity signal on T2 weighted images, homogeneously enhancing post-gadolinium, as well as absence of high-signal intensity from the posterior lobe on T1. Neither of the imaging findings is highly specific and allows to make a correct diagnosis (Schubiger and Haller 1992). The definitive diagnosis of metastatic involvement is always based on histological evaluation, allowing to distinguish from pituitary primary lesions (Go et al. 2011).

Benign lesions more often are functioning masses, frequently producing ACTH and prolactin; on the other hand, metastases might cause disfunction of the pituitary gland and compression of the near anatomic structures (Max et al. 1981).

Treatment, mostly palliative, depends on the symptoms. Surgical exploration and decompression, alone or combined with radiation, is often necessary when suprasellar extension causes progressive deterioration in vision and/or pain (Branch and Laws 1987). In our case, tumor debulking allowed to improve the local symptoms, especially headache and visual field defects such as diplopia and hemianopsia.

Generally, the surgical approach, the completeness of the resection, and an aggressive treatment (surgery plus local radiation) are associated with better symptom relief but do not affect survival rates; in fact, prognosis of patients with MPs is usually poor, with a life expectancy of few months after diagnosis (Komninos et al. 2004).

The peculiarity of the case described above is not only represented by the unusual site of metastasis from rectal cancer, but also by the long history of treatments received by our patient.

Recent studies show that the expanded treatment options and the consequent improved survival for patients with metastatic CRC are associated with an increased incidence of metastases at uncommon sites (Sundermeyer et al. 2005). A comprehensive review on the incidence, prevalence, epidemiology, risk factors, management, and prognosis of brain metastases arising from esophageal, gastric, gallbladder, pancreatic, small bowel, and colorectal cancer reported that brain metastases are found in 1% of colorectal cancer, 1.2% of esophageal cancer, 0.62% of gastric cancer, and 0.33% of pancreatic cancer cases. Survival in patients with brain metastases from gastrointestinal tumors was found to be inferior compared with breast, lung or kidney. Authors concluded that, although early treatment has been linked to prolonged survival and improved quality of life, brain metastases represent a late manifestation of gastrointestinal cancers and remain an ominous sign (Go et al. 2011).

Sundermeyer and colleagues evaluated patients with metastatic CRC from 1993 to 2002 at the Fox Chase Cancer Center, collecting date of diagnosis/metastasis, primary tumor site, therapeutic agents received, survival, and site(s) of metastases. The data demonstrate that the incidence of bone and brain metastases in patients with CRC is more common than previously reported and is associated with the administration of multiple systemic treatments (Sundermeyer et al. 2005).

In conclusion, here we presented the case of a patient affected by metastatic rectal cancer, with a large sellar mass and a long history of oncological treatments.

The rarity of involvement of the pituitary gland from rectal cancer made diagnosis challenging and the histological evaluation helped to confirm the intestinal origin of the lesion. This metastatic event is very uncommon making almost impossible to perform prospective clinical trials specifically designed to compare different treatment approaches. Thus, only a greater awareness of the problem along with a more accurate and timely diagnosis, will lead to choose the best therapies suitable to the specific patient and in turn improve overall prognosis.

Although infrequently, metastatic involvement of the pituitary gland from colorectal cancer can be found and it might become increasingly common in the next future as a result of the expanded treatment options and the consequent improved patients’ survival.
